# Older people’s priorities in health and social care research and practice: a public engagement workshop

**DOI:** 10.1186/s40900-016-0016-0

**Published:** 2016-02-06

**Authors:** Dalal Alsaeed, Nathan Davies, Julia Fiona-Maree Gilmartin, Elizabeth Jamieson, Kalpa Kharicha, Ann E. M. Liljas, Bahijja Tolulope Raimi-Abraham, Janine Aldridge, Felicity J. Smith, Kate Walters, Mine Orlu Gul

**Affiliations:** 1 University College London, School of Pharmacy, 29-39 Brunswick Square, London, WC1N 1AX, UK; 2 University College London, Institute of Epidemiology & Health, Primary Care & Population Health, Royal Free Campus, Rowland Hill Street, London, NW3 2PF, UK; 3 Age UK London, Greater London Forum for Older People, 1st Floor, 21 St Georges Road, London, SE1 6ES, UK; 4 Centre for Medicine Use and Safety, Faculty of Pharmacy and Pharmaceutical Sciences, Monash University, Melbourne, Australia

**Keywords:** Public engagement, Older people, Age inequality, Involvement, Research

## Abstract

**Plain English Summary:**

A one day public engagement workshop was held to focus on the priorities of older people about research and practice in health and social care. Seventy-five older people from the general public and a variety of backgrounds attended this event to share their views and discuss what should be prioritised. The main aim of this workshop was to identify and prioritise issues that are important to older people that would benefit from further research, as well as create an environment for older people to share ideas and problems related to these important issues. Key priorities brought up by participants included loneliness and isolation, support and training for professional and family carers, post-surgical care, negative perceptions of older people and inequalities related to public services and healthcare. Participants further suggested older people should be actively involved in all stages of the research process.

**Abstract:**

As the world’s population ages, there is an increasing need for research that addresses the priorities of older people. A public engagement workshop focusing on the priorities of older people for research and practice in health and social care was attended by seventy-five people aged 70 years and above in London, United Kingdom (UK). The workshop aimed to identify and prioritise issues important to older people that would benefit from further research and act as a platform to promote sharing of ideas and problems related to these important issues. Key priorities emerged including loneliness and isolation, support and training for professional and family carers, post-surgical care, negative perceptions of older people and inequalities related to public services and healthcare. Participants further suggested older people should be actively involved in all stages of the research process.

## Background

The importance of involving the public in health and social research, particularly in policy development, has been recognised internationally [1]. A recent systematic review on the impact of patient and public involvement demonstrated the importance of involving the public in health research to ensure that it is relevant and reaches service users [2]. It has been advocated that health services should be ‘patient-led’ and encourage public trust [3].

In mid-2014, there were over 11.4 million people aged at least 65 years old in the UK, constituting 17.7 % of the total population, with a projected total of over 20 million by 2032 [4, 5]. This ageing population has resulted in a greater proportion of older people (aged 65 and above) requiring care and support, and has led to a more focused effort in recent years to enhance ageing healthcare policies. The challenges of longer life can be addressed by exploring ways to keep people healthier, happier and independent for longer; to remove discriminatory barriers for older people, and to support people with disabilities, long term health conditions and/or those needing care, in times of economic austerity [6].

It is also becoming increasingly important for older people’s experiences and opinions to be considered when conducting research [7]. The involvement of older people in research is likely to generate new knowledge and understanding of their lives, as well as have the potential to shape policies, practices and services that might contribute to their well-being.

Previous priority setting exercises designed to identify older people’s issues have specifically addressed factors relating to medicines, such as barriers to their use, and how to improve their provision [8, 9]. However, these exercises have not considered what older people view as important research questions in ageing in healthcare and in a broader sense. The published literature includes data obtained from interviews and focus groups regarding older people’s opinions on ways to provide person-centred support [10] and the feasibility of their involvement as co-researchers [11, 12]. Similarly to previously published studies, the current workshop aimed to engage a sample of older people in dynamic discussions to inform future research, with a specific focus on important topics that require further attention.

## Methods

A workshop titled ‘What are the important questions for research in ageing?’ was held at the University College London (UCL) School of Pharmacy in April 2014, to understand issues pertinent to older people in the pursuit of facilitating patient-centred care and to address increased public involvement in research on ageing.

Participants were contacted and invited to attend the workshop via a mailing list from a local voluntary sector organisation, Age UK London. This was conducted through a representative from the organisation in order to preserve confidentiality. Seventy-five older people attended the workshop. Participants were informed at the beginning of the workshop that this was a discussion to gather their views on age inequalities and active involvement of older people in research and how the information they provided would be used.

The topics discussed included personal motivation in attending the workshop, how older people could be more actively involved in research, priorities for research involving older people and health care, as well as experiences and attitudes towards age inequalities and strategies to address them. Following brief introductory talks, participants worked in 9 small groups of up to nine people. The small groups were facilitated by nine researchers who were experienced in group facilitation. Facilitators recorded all the ideas and discussions that were shared in their small groups contemporaneously. Following group work, ideas were shared with the larger group. Facilitator notes from the small group discussions and ideas and suggestions provided by the participants on the day were collated and categorised thematically by MO and DA. The research team was diverse in its expertise and perspectives brought to this agenda, and the findings were discussed by all the researchers to increase the rigour of data analysis.

## Result

Discussions were centred around three priori themes (motivation to participate, active involvement of older people in research, and age inequalities). Three sub-themes emerged from the discussions around age inequality: negative perceptions of older people, having a strong voice, accessibility of services and support, and age inequalities in healthcare. The findings are presented below.

### Motivation to participate

Participants shared their reasons for attending the workshop, which included the desire to gain new knowledge, in particular relating to ensuring their rights for certain health and social care services were upheld, and represent an older people’s forum and give a voice to older people’s perspectives.

### Active involvement of older people in research

Participants felt that there was much to gain from their longer life experience compared with younger age groups and were extremely enthusiastic and willing to offer support in the area of research involving older people. Participants expressed a desire to be involved in creating solutions to healthcare and other issues concerning their age group, as per the phrase “Nothing about me without me” from the UK Department of Health report on promoting equity [13]. Participants expressed an interest in being involved in biomedical research and hoped that the findings of such studies would be shared with Government authorities and ‘think tanks’. Participants also believed that they should be involved in every stage of research, from involvement in grant applications, to dissemination of research findings. They also expressed a desire for older people to be integrated with younger generations, such as volunteering with local authorities to contribute to the community, rather than being viewed as a ‘separate community’.

Various suggestions for the active involvement of older people in research were discussed, such as involvement in doctoral projects, clinical studies, and patient panels at general practitioner (GP) surgeries. Participants felt that the involvement of older people in these areas could play a pivotal role in the planning of healthcare services, social care and related policies.

### Priorities for research in ageing and health and social care

Participants identified a range of priorities that they felt were important areas for research and these mostly related to areas where they felt there were deficiencies in current health and social care. The topic of isolation and loneliness was felt by many to be of great importance and participants felt more awareness should be raised to deal with this issue. They highlighted a need to prioritise research on age-related conditions that have a negative impact on quality of life. In some groups improving post-surgical care at hospitals was discussed, for example, the transition period between the hospital and the patient’s home.

Concern regarding support and training for both professional and family carers was also raised. Participants mentioned the need for improved training for carers. The minimal support received by family carers was regarded as unfair, and further research to examine the burden on family carers and how best to support them was considered important.

### Age inequalities

A number of themes related to age inequalities were identified during the discussions and are described below.

#### Negative perceptions of older people

Many participants felt that society held negative perceptions of older people and they expressed a wish for this negative image to change. Participants emphasised their valuable contributions to society and explained that they should not be considered a ‘problem’ to society. Most of the participants felt that they were perceived negatively by the public and the media. Participants felt that they were an unacknowledged resource that was regularly used. For example, participants explained that many older people perform unpaid carer duties, are active grandparents, and are organisational volunteers. The issues of labelling older people and stereotyping them based on their medical conditions were also raised by participants.

#### Having a strong voice

Participants reported finding it difficult to access and approach governing bodies and felt that they are not given the opportunity to share their thoughts and opinions. As one participant stated, they have “*the right to be heard and the right to a response*”. Participants felt that governing bodies and policy makers need to listen carefully and implement solutions to problems based on what they have heard from older people, for effective outcomes. It was also suggested that a minister should be appointed to specifically represent the older population.

#### Accessibility of services and support

Older people often felt that information, public services and support are sometimes inaccessible to them due to their age. For example, older people who may not have access to a computer, or do not know how to use one, may be restricted from accessing information that is only available online, or from completing on-line applications to access services relating to public transport, retirement, utility bills and insurance. As a result, it was concluded that technology, on its own, cannot be considered a viable solution to societal problems if not all members of the community can use it.

Participants also wished to integrate medical and social services to improve their accessibility, such as giving individualised information to older people on a regular basis, concerning the services available to them and alternative treatments.

#### Age inequalities in healthcare

Age inequalities featured heavily in the workshop discussions, and it was made evident that inequalities not only existed between the young and old, but also within the older population. Older people suggested that they require longer GP appointments than younger patients, to accommodate their multiple co-morbidities and ailments. It was felt that there were barriers to accessing care at GP clinics. For example, receptionists may be able to make decisions regarding who can and cannot see the GP and older people may not be able to access a GP who has experience in treating older people. Participants also felt that rationing of healthcare caused age inequalities, with age-related conditions that impact considerably on their lives such as cataracts and knee pain considered high priority issues by older people, but potentially seen as low priority by healthcare providers. Some participants felt that GPs discriminated against them when they refused referral to specialists for older age-related complaints. Participants explained that the phrase “‘*it*’*s your age*” was frequently used by healthcare professionals to explain away aches and pains.

Some participants felt that the treatment and screening of certain diseases based on age was unfair, such as screening for bowel cancer, which in the UK is only routinely conducted in people aged between 60–74 years old [14]. It was also suggested to extend the cut-off age for breast cancer screening.

Additional issues in which participants felt that they were treated differently to younger individuals included the dispensing of medicines. Participants also felt that certain resources for older people, such as podiatrists, hearing aids and shingles vaccinations, were inadequate.

## Discussion

As the world’s population ages, concerns and issues related to older people will challenge health and social care professionals, policy makers and consumers alike. The involvement of older people in all stages of research is important to identify and prioritise relevant areas of research. The participants at this workshop believed that they could contribute to research and expressed a willingness to be included in all stages of the research process.

Many of the participants were particularly keen that research should address issues of loneliness and social isolation in older age as a priority. Other suggestions for research in ageing and health and social care discussed were those relating to age-related conditions and their effects on quality of life. Health policy is moving towards a more integrated model, which participants voiced as essential to improve the accessibility of health and social services.

The published literature has demonstrated the high motivation of older people to be involved in research and decisions concerning them [10]. Older people have also been involved in research as co-researchers, producing targeted findings and benefiting all those involved [11, 12]. Older people have expressed the desire to be viewed as individuals who each have their own set of priorities and views [10]. A previous study has demonstrated that issues surrounding health were considered of highest importance to older people, followed by issues relating to transport and housing [15]. These issues are similar to those raised in the discussions in the current workshop. Furthermore, participants wished to have their opinions heard and implemented, so that tailored services for older people could be produced.

Evidence of age discrimination in policy and practice in primary health care in the UK was found in a review by the Centre for Policy on Ageing (CPA) in 2009. Examples of age discrimination included: age-limits on disease screening; limited specialist referrals; under treatment (compared to younger people); receiving negative attitudes from healthcare professionals; barriers to accessing services; and limited services that meet their needs [16]. Our findings are comparable to these in that workshop participants reported feeling stereotyped by healthcare professionals, as well as feeling as though they were taken less seriously and treated unfairly due to their age.

It has been reported that older people are treated and referred less than their younger counterparts in a range of health areas, including stroke prevention [17], referrals for hip pain and stomach pain [18], hip and knee replacement [19] and cognitive behavioural therapy for depression and anxiety [20]. In some cases this may be justified, for example when the risks of surgery are greater than the benefits, but in other cases participants felt that this may be considered unfair. It is often unknown whether these differences are justified and the potential reasons for these differences are wide and varied. Future research could consider exploring whether older people want specific healthcare services adapted to their needs, whether older people have difficulties in accessing services, whether services may be more harmful for older people compared to younger individuals and if professionals are being ‘ageist’. Research of this nature could enable the implementation of tailored solutions and the reduction of any age inequities that may potentially exist in the provision of healthcare.

Our research has confirmed some of the findings of previous studies such as older people’s willingness to be involved in research, including active participation and contributing to all stages of research. Our engagement day identified key priorities in ageing which require research and action. Some of these have been identified previously but there is still a need for these to be further addressed: isolation and loneliness, age-related chronic conditions that impair quality of life, post-surgical care and age inequalities in health and social care. With respect to medicines, the workshop highlighted several priorities including the valuable role that family carers play in all medicines-related and social care aspects and the need for carer support; the preferences of older people for different medicine formulations; and engagement with health care professionals. As a consequence, these topics have now been included into our research agenda.

### Limitations

Although the participants were all aged 70 years or older, they were recruited from one organisation in London and their detailed demographic data was not collected. Participants had to be able to attend the venue (which was accessible), and participate in English. This may mean we have missed issues that are pertinent for groups of individuals who have different and specific needs; for example, non-English speaking individuals, those with more advanced dementia and those unable to leave their homes. We also have no information on those who declined to participate. We do not know how well our participants represent the diversity in the community, and may not represent the views of older people who did not attend. However, it is a strength of our study that participants represented a range of different ethnic groups, with a variety of health conditions and disabilities, leading to a wide range of views being expressed.

## Conclusions

The workshop enabled older people to provide their perspectives on possible ways of actively participating in research. Participants strongly felt that older people should be at the heart of ageing research and actively involved in all stages of the research process. They identified key topics in ageing to be addressed: including isolation and loneliness, support for carers, age-related chronic conditions that impair quality of life, post-surgical care and age inequalities in health and social care. There was a perception that age inequalities exist in health and social care; including access to services and information due to peoples’ inability to be able to make use of technology, inadequate consultation times with primary care physicians, disease screening, and the negative perception of older people by society. We have embarked on research that addresses key topics raised at this event, for example, needs of carers who support older people in their use of medicines, preferences for and management of different medicine formulations, and working with health professionals to raise awareness of these issues and how they may be more effectively addressed. Future research projects could seek involvement from representatives of UK organisations that represent older people. This could facilitate dissemination of research findings both locally and nationally, and beyond the scientific community. More targeted input around specific research areas could be achieved by focussing on a few issues, such as whether healthcare services are adapted to older people’s needs and whether older people have difficulties in accessing such services.

## Box 1: What this paper adds

**Table:** 

What is already known:
• Issues surrounding health were considered of highest importance to older people • Concerns of older people about age discrimination in policy and practice in primary health care in the UK • Older people are keen to participate in research and have a voice in planning of services
Our study provides evidence that older people are:
• Keen that research should address issues of loneliness and social isolation in older age as a priority • Keen to dispel what they see as negative perceptions of older people by the wider community and wish to highlight the benefits of their experience and be recognised for the unpaid services they often provide • Aware of the important role of the family carer in medicines-related and social care aspects and believe that research should be undertaken as to how best to support them
